# Experiences of Full Body Skin Examinations among Sexual and Gender Minority Patients in Dermatology: A Qualitative Study

**DOI:** 10.21203/rs.3.rs-10339621/v1

**Published:** 2026-08-03

**Authors:** Natalia N. Khosla, Robin Z. Ji, Michelle Verghese, Jincong Q. Freeman, Adena E. Rosenblatt

**Affiliations:** The University of Chicago; The University of Chicago; The University of Chicago; The University of Chicago; The University of Chicago

**Keywords:** Health disparities, anticipatory stigma, full body skin exam, sexual and gender minorities, minority stress, medical mistrust

## Abstract

Sexual and gender minority (SGM) patients experience disproportionate skin cancer risk and barriers to preventive dermatologic care access relative to non-SGM counterparts. Yet SGM may be deterred from the full body skin exam (FBSE) for screening due to fear of stigma surrounding their bodies. There is a dearth of qualitative research about SGM patients’ perceptions of the FBSE.

To address this gap, we surveyed 175 SGM-identifying patients; 161 provided qualitative responses describing whether their sexual or gender identity (SOGI) influences their decision to undergo a FBSE and why.

We identified pervasive themes of discomfort, fear of clinician discrimination (anticipatory stigma), and prior discriminatory experiences. The 69.6% of participants who reported their SOGI did not influence their decision to undergo a FBSE most commonly described enduring the FBSE for health benefits despite significant discomfort. Among the remaining 30.4% of participants, discomfort was substantial enough to influence their decision to undergo a FBSE, with 75.5% citing fear of clinician discrimination as a cause for hesitation.

Forty-six participants provided specific recommendations to improve the FBSE experience for SGM, including: providing gowns and chaperones, visible displays of allyship (e.g. flags), offering SGM-specific care (e.g. for top surgery scars), affirming communication (correct pronoun use and avoiding anatomy assumptions), eliciting trauma history, and continuous consent throughout the exam.

Across responses, a unifying theme was that clinicians’ lack of understanding about SGM experiences contributed substantially to patient discomfort.

Our findings suggest that fear of clinician discrimination and past discriminatory experiences may discourage some SGM patients from undergoing FBSEs, which may exacerbate existing health disparities burdening this population. These findings underscore the need for SGM-specific education and bias reduction in dermatologic training.

## INTRODUCTION

Sexual and gender minority (SGM) patients experience disproportionate disease burden relative to non-SGM counterparts, including higher rates of chronic health conditions [1], earlier disability onset [2], greater substance use and mental health disorders [3, 4], and lower likelihood of receiving cancer prevention [5]. These disparities stem from health risk factors, medical discrimination, under-insurance, and minority stress–the chronic stress produced from experiencing prejudice [6].

SGM patients experience higher rates of skin cancer than non-SGM patients [7], with gender-nonconforming men having double the adjusted odds of skin cancer compared to cisgender men [8]. The full-body skin exam (FBSE) is a widely-used and highly effective head-to-toe visual examination to detect early signs of melanoma. However, SGM patients may be less likely to be offered screening and may avoid the exam due to fear of stigma around their bodies [9, 10].

Given sparse data on SGM patients’ attitudes towards the FBSE, we surveyed this population to identify areas for improvement in SGM dermatologic care.

## MATERIALS AND METHODS

Between June 2022 and April 2023, we conducted an anonymous survey of SGM-identifying adults (18 + years) via REDCap at the University of Chicago; quantitative results have been published previously [11]. The study was IRB-approved (#22–0498). Of 206 participants, 175 completed the survey and provided sexual or gender identity (SOGI) information.

Quantitative data were analyzed using *t*-tests, Wilcoxon rank-sum, Pearson’s Chi-squared, or Fisher’s exact tests as appropriate. For qualitative data, two coders independently developed themes, subthemes, and a codebook, then reconciled differences iteratively until thematic saturation and consensus were reached. The finalized codebook was applied to all responses and final codes were analyzed. Descriptive analyses were performed using Stata 18 (StataCorp LLC).

## RESULTS

Of 175 survey respondents, the mean age was 32.7 years (SD 12.3), 60.6% identified as non-Hispanic White, and 42.9% reported post-graduate education. Overall, 54.4% self-identified as lesbian or gay, 24.6% as queer, pansexual, questioning or other, 17.5% as bisexual; 39.8% self-identified as cisgender male, 24.1% as cisgender female, 19.3% as gender queer, non-conforming, or non-binary, and 16.9% as transgender.

### Influence of SOGI on FBSE Decision

Of 161 respondents to, “Does your gender identity or sexual orientation influence your decision to have a full body skin exam? Why or why not?”, 112 (69.6%) said no, their SOGI does not influence their decision to obtain an FBSE; 49 (30.4%) reported that yes, it does.

Despite this difference, seven out of eight major themes emerged across both groups, notably provider-specific factors influencing comfort (including preference for same-gender or LGBTQ+ providers) and discomfort with the exam or providers (Table 1).

No: SOGI does not influence FBSE decision

Among “no” respondents, top themes were: 1) prioritization of health (46.4%), 2) perceived irrelevance of gender/sexual identity to health (33.0%), and 3) provider-specific factors influencing comfort (25.0%). Notably, discomfort was frequently reported in responses about prioritizing potential health benefits of the FBSE, reflecting a willingness to endure the exam despite discomfort: *“I recognize it’s importance so go through it even if it is emotionally hard. Honestly may take anxiety meds before.”*

Yes: SOGI does influence FBSE decision

Among “yes” respondents, the top themes were: 1) experience/fear of providers’ discrimination or judgment (75.5%), 2) provider-specific factors influencing comfort (57.1%), and 3) experience/fear of societal bias & power dynamics (24.5%). Subthemes included fear of non-affirming language, pessimism about provider bias, and fear of judgment of trans anatomy: “*I’ve encountered enough homophobia in… the medical world that I would be hesitant to be vulnerable like that with a doctor I felt was potentially homophobic.”*

### Patient Suggestions for Improvement

Forty-six participants responded to the question: “What can medical providers and staff do to improve the experience of a full body skin exam for LGBTQ+ patients?” The top themes were: communication & consent (56.1%), creating a safe and inclusive environment (43.9%), and reducing anxiety and judgment (24.4%).

The overarching theme was that clinicians” lack of knowledge of SGM experiences contributes to patient discomfort. Suggestions for improvement included:

1. The clinical environment

• Provide gowns and chaperones

• Incorporate visible displays of inclusivity (flags)

• Include and use gender identity on intake forms

• Employ more queer physicians

• Let patients meet and choose clinicians before care

2. General tips

• Avoid inappropriate questions about sexual orientation when irrelevant

• Use correct pronouns and and avoid triggering language (e.g. “lay down”)

• Be educated on basics (e.g. trans terminology, common procedures)

• Do not make assumptions about anatomy from physical appearance

• Offer SGM-specific care (e.g. top surgery scar care)

• Recognize and reduce patient’s anxiety

• Treat SGM patients with equal care as non-SGM

3. Before the exam

• Ask relevant questions about gender and sexuality while dressed

• Do not put the burden on patient to bring up gender or sexuality if relevant

• Elicit patient concerns to determine what is relevant to ask

• Elicit trauma or trigger history

• Ask permission instead of telling the patient what will be done

4. During the exam

• Continuous consent (not “one and done”)

• Keep conversation flowing

• Narrate rationale for each step before *and* during touch

• Conduct structured, systematic history and exam

• Re-affirm allyship

5. Physician education

• Trauma informed care and gender dysphoria

• Familiarity with SGM life experiences

## DISCUSSION

In this qualitative study, we identified pervasive themes of discomfort, fear of clinician discrimination, and past discriminatory experiences among SGM patients in dermatologic care. For 30% of participants, these concerns were profound enough to influence their decision to undergo a FBSE. To our knowledge, this is the first qualitative study to examine how anticipatory stigma and past experiences with clinician discrimination may discourage SGM patients from seeking skin cancer screening.

Many participants who reported that their SOGI does not influence their decision to get a FBSE described undergoing FBSEs despite substantial discomfort because they prioritized the health benefits of screening. Conversely, participants who reported that their SOGI does influence their decision to get a FBSE reported discomfort severe enough to discourage them from undergoing a FBSE. Notably, 75% of this group expressed either fear of physician discrimination or past discriminatory experiences as reasons for their hesitation to undergo a FBSE.

These findings are consistent with a growing medical literature about the deleterious consequences of medical and societal bias for minority groups: including minority stress, medical mistrust, and anticipatory stigma driving care avoidance, thereby compounding health disparities. Anticipatory stigma is associated with reduced ART adherence among Black women with HIV [12], healthcare avoidance by transgender patients [13], and delayed care seeking by veterans with depression [14].

Participants identified concrete interventions for improving FBSEs for SGM that dermatologists should implement. These included providing gowns and chaperones in clinic, visible displays of allyship (e.g. flags), employing LGBTQ + or visibly allied clinicians, offering SGM-specific procedures (e.g. for top surgery scars), avoiding anatomy assumptions, using preferred pronouns, eliciting trauma history, and continuous consent with narration during exams. Patients consistently emphasized the need for improved physician education about SGM health: a major area of opportunity, given that 46% of dermatology programs in 2020 included zero hours of SGM-specific education [15].

Limitations to this work included lack of non-SGM control and the cross-sectional, observational design. Future studies should take a prospective approach with non-SGM control groups to better identify potential causal associations.

## Supplementary Material

This is a list of supplementary files associated with this preprint. Click to download.

• SupplementaryFigure.docx

## Figures and Tables

**Table 1:** Question 1 subthemes and exemplary quotes. *“Does your gender identity or sexual orientation influence your decision to have a full body skin exam? Why or why not?”*

Themes	Subthemes	Frequency	Percent
**1. Discomfort with the exam and/or providers unrelated to SOGI**	**a. General discomfort with physical exposure (e.g., feeling uncomfortable being naked in front of a medical provider)** *“I feel uncomfortable when naked in front of a medical provider and I don’t think I’d feel differently if I were a different gender or sexual orientation.”* *“It doesn’t make a difference just showing my genitalia to people is uncomfortable ”*	8	7.1%
	**b. Discomfort related to body image or self-consciousness (e.g., not 100% comfortable with body due to body hair, eating disorder history, etc)** *“My hesitation would stem more from body image/eating disorder history and being seen naked rather than my sexual orientation. I would consent if 1) it was medically necessary and 2) I trusted the provider (this would include them being accepting of my sexuality if they had been made aware of it). ”*	5	4.5%
		13	**11.6%**
**2. Provider-specific factors influence comfort**	**a. Preference for same-gender physician (e.g., feeling more comfortable with a female doctor as a woman)** *“If it seemed necessary I’d do it: prefer same-gender physician”* *“I am a (bisexual) cisgender female, I always choose female doctors. If my doctor was a male, I would be more uncomfortable with the undressing required for the exam.”* *“My sexual orientation does not impact how I feel about the exam. The full body exam is more relevant to my gender. I am a woman slightly more uncomfortable with a man examining me, but I am still relatively comfortable and open with it.”* *“I’m comfortable having a full body exam, though I’d prefer the same gender performing the exam.”*	9	8.0%
	**b. Preference for LGBTQ+ physician** *“Previous dermatologist was gay. There was a stronger connection and ease”*	1	0.9%
	**c. Importance of an established familiarity/trust with the physician** *“Has not been a negative factor with the doctor conducting the exam - trust was already established.”*	6	5.4%
	**d. Preference for providers who are affirming, allies, informed on SOGI** *“Because it does not impact my risk of skin cancer. I’m more concerned about the doctor being trauma informed and willing to work with me on my comfort level of monitoring vs biopsy”* *“the dermatologist office should has extensive experience working with the LGBT community”*	5	4.5%
	**e. Valuing physicians who demonstrate professionalism/respect in their attitude (i.e. nonjudgmental)** *“Not 100% comfortable with my body or skin (e.g. I never shave), but okay for an exam if the doctors are not judgemental.”*	7	6.2%
		28	**25.0%**
**3. Trust/comfort with self, exam, and/or providers**	**a. Trust in the medical system and the professionalism of the physician** *“Im comfortable with my body and feel that I my doctor will be professional and do the exam without any sort of bias. However, I do a lot of research on which doctors/providers I go to prior to making an appointment. If it was a randomly chosen provider, I’d be more hesitant.”* *“trust doctors’ professional judgement”*	8	7.1%
	**b. Self-assurance in body, gender identity, sexual orientation during the exam** *“I’m comfortable with the process and my identity.”* *“I am comfortable having my body examined”* *“I’m old as dirt and love showing what’s left of my body after 35+years of HIV”*	19	17.0%
		27	**24.1%**
**4. Perceived irrelevance of gender/sexual identity to health**	**a. Belief that identity is irrelevant to the decision to have a TBSE** *“I can’t think of a specific way my gender or sexual orientation impact my dermatological health.”* *“I’m fine with getting a full body skin exam and it has nothing to do with my sexual orientation ”*	25	22.3%
	**b. Never considered identity in the decision-making process (e.g., I never thought about the relevance of identity to the exam)** *“I never thought about it”*	7	6.2%
	**c. Skepticism and/or opposition to linking identity to medical care** *“Because the gender identity, the sexual orientation and the gender expression are irrelevant to my skin health. All those three characteristics above mention are part of people’s private life. No one, nor researchers nor law makers nor politicians should ever enter in those spheres to ask nor to legislate about them. Leave LGBTQ+ and BIPOC people alone. This research and the laws are way too invasive. Stop it.”* *“I don’t think that should be part of the conversation for medical care”* *“Because I don’t your sexuality should determine your health”*	5	4.5%
		37	**33.0%**
**5. Prioritization of health**	**a. Willingness to undergo the exam despite discomfort** *“I recognize it’s importance so go through it even if it is emotionally hard. Honestly may take anxiety meds before.”* *“My health must come first no matter how uncomfortable.”* *“I prioritize taking care of my health even when it is uncomfortable. My mom had melanoma so I really want to stay up to date with my skin exams. ”* *“I do sometimes have awkward encounters with medical providers when they see my top surgery scars and don’t know what they are, but my concerns for my own health override any concerns about being treated differently by providers.”* *“While it might be uncomfortable, I think it is important to be informed about my health regardless!”*	11	9.8%
	**b. Motivated by the medical necessity of the exam (e.g., family history of skin cancer)** *“I don’t want skin cancer.”* *“Important for early detection of skin cancer”*	29	25.9%
	**c. Accepting the exam as a routine health practice / necessary medical procedure** *“I think the scan is very important.”*	12	10.7%
		52	**46.4%**
**6. Influence of past experiences with providers/health care**	**a. Positive past experiences with providers** *“I have eczema, and I have had really good experiences with my dermatologist with examinations and felt very respected. ”*	6	5.4%
	**b. Negative past experiences with providers** *“I do sometimes have awkward encounters with medical providers when they see my top surgery scars and don’t know what they are, but my concerns for my own health override any concerns about being treated differently by providers.”*	2	1.8%
		8	**7.1%**
**7. Experience/fear of providers’ discrimination or judgment against SGM**	**a. Fear of non-affirming language, behavior, or misgendering (e.g., harassment)** *“I would be nervous about being misgendered by a new doctor.”*	1	0.9%
	**b. Experiences/fear or pessimism about medical providers being biased based on SOGI** *“I said no but it could, depending on circumstances. My dentist was the one who treated me negatively I think because of his perception of my sexuality. If a doctor was like that I’d want to avoid him or her.”*	1		0.9%
	**c. Experiences/fear of judgment of trans anatomy** ***“The fact that I am transgender and have a different anatomy from other people perceived as women would definitely make me hesitant to undergo a full-body skin exam, and I would want to talk about the issue with my provider first.”***	2	1.8%
		4	**3.6%**
**8. Experience/fear of societal bias & power dynamics against SGM**	**a. Experiences or awareness of societal stigmatization of sexual/gender minorities** *“I believe doctors should be understanding no mater of the situation. But I’m also a cis white gay man and I do understand that everyone has different experiences ”*	4	3.6%
	**b. Experiences or awareness of societal misogyny and/or sexism** *“Less my orientation and more just my general discomfort with having someone look at my body (related to internalized homophobia and misogyny though). I would absolutely refuse to be examined by a male doctor and wouldn’t be all that comfortable with a female doctor either (I am female for reference)”* *“It feels difficult to separate issues with my gender versus with my sexuality, as in, being a woman experiencing sexualization and discomfort due to the way my body is discussed and perceived by others is centrally where my discomfort is.”*	2	1.8%
		6	**5.4%**
Themes -	Subthemes	Frequency	Percent
**1. Discomfort with the exam and/or providers related to SOGI**	**a. Discomfort with genitals being visible/nudity** *“If told I needed one I would consent, but I tend to be uncomfortable with doctors and especially showing intimate body parts to doctors.”*	5	10.2%
	**b. Discomfort related to gender dysphoria** *“Gender dysphoria makes me go less often than I otherwise would.”*	6	12.2%
		11	**22.4%**
**2. Provider-specific factors influence comfort**	**a. Preference for providers who are LGBTQ+** *“if i wasn’t comfortable with my doctor and knew they weren’t specifically queer, or queer friendly i wouldn’t be comfortable ”*	4	8.2%
	**b. Preference for same-gender or nonbinary providers** *“I would only be comfortable with a full body skin exam if offered by a female or nonbinary (in the umbrella term sense) provider - my experiences as a woman would make me uncomfortable receiving a full body exam from a male doctor.”*	4	8.2%
	**c. Discomfort with male providers** *“As a gay cis male, I’m uncomfortable having a male dermatologist perform my exam”* *“i would be uncomfortable undressing in front of a male doctor”*	5	10.2%
	**d. Importance of building trust with the provider (e.g., needing to establish a relationship before feeling comfortable)** *“I’m more nervous and shy because of my genitals. I would have to build a relationship with a provider to feels comfortable. ”*	2	4.1%
	**e. Desire for providers who are affirming, allies, and informed on SOGI** *“I’m transmasc non-binary and on testosterone, so my body is different from the norm and I’m still adjusting and getting comfortable. It helps that my dermatologist is a gay man and super affirming-if I needed it done, I’d be glad it’s with my current dermatologist.”* *“If it is a gay doctor or a doctor I have vetted as affirming or an ally, I will do it. I don’t want to do it with a new doctor.”* *“If the provider is not inclusive and informed I would not consent. ”*	9	18.4%
	**f. Desire for providers who make an effort to create a safe environment** *“it depends on the facility and efforts to be sensitive.* *if there is little effort, it is harder to make the decision”*	4	8.2%
		28	**57.1%**
**3. Attempts made to protect self from provider bias**	**a. Hiding gender/sexual identity from providers** *“I am trans and my doctor doesn’t know. This is a conversation I don’t feel comfortable/ want to have with a doctor that I don’t know is an ally”*	2	4.1%
	**b. Researching/vetting providers for inclusivity** *“I would be open to it but my gender identity would cause me to need to look for an inclusive place, otherwise I would not have it done. ”*	2	4.1%
		4	**8.2%**
**4. Perceived relevance of gender/sexual identity to health**	**a. Belief that identity may influence health decisions (e.g., sexual orientation may affect skin diseases)** *“sexual orientation may affect skin diseases”*	3	6.1%
		3	**6.1%**
**5. Prioritization of health**	**a. Willingness to undergo the exam despite discomfort** *“Though I would undergo the exam in order to mitigate the risk of skin cancer, I would be hesitant that my care provider would judge my body”*	6	12.2%
		6	**12.2%**
**6. Influence of past experiences with health care**	**a. Negative past experiences with health care** *“I’ve become less trusting of my doctors. Also I’ve felt like they were looking at me bc of my identity and not bc of what I was telling them. After learning about me they didn’t adjust themselves to fit me they just treated me the same without considering my needs.”* *“I don’t experience affirmed care in many interactions with medical professionals”* *“I was nervous to have one because of previous bad experiences with medical professionals.”*	8	16.3%
		8	**16.3%**
**7. Experience/ fear of discrimination from medical providers**	**a. Experience/fear of non-affirming language, behavior, or being misgendered (e.g., harassment)** *“I am a little more hesitant because I am nonbinary and I am not always confident that the provider will be gender-affirming. I have taken testosterone for many years but I haven’t had surgeries, so in some ways, my body still looks like what might be perceived as a cis woman’s body.”*	12	24.5%
	**b. Experience/fear of physical discomfort or judgment** *“I feel judged sometimes”*	5	10.2%
	**c. Experience/fear or pessimism about medical providers being biased based on SOGI** *“I’m gay and pretty visibly queer- being naked for a full body exam is definitely a vulnerable spot, and I’ve encountered enough homophobia in the real world and in the medical world that I would be hesitant to be vulnerable like that with a doctor I felt was potentially homophobic or who I didn’t feel comfortable being out/being myself around”* *“Being afraid of potential discrimination by doctors”*	12	24.5%
	**d. Experience/fear of judgment of trans anatomy** *“because cis people are generally uncomfortable with unexpected genitals for whatever reason.”* *“Difficult to try to find a trans-affirming dermatologist that will not ask about my top surgery scars unnecessarily”*	8	16.3%
		
		37	**75.5%**
**8. Experience/ fear of societal bias and/or power dynamics against SGM**	**a. Voyeurism/fetishization of gender minorities** *“Carrying the shame of having a non male body + the fetishization of lesbianism, along with the frequency of physicians who are white men that symbolize violence to me often.”*	3	6.1%
	**b. Societal stigmatization of sexual/gender minorities** *“judgment on how i present my gender”*	4	8.2%
	**c. Doctor & provider power/privilege over patient** *“Having had discriminatory and related painful experiences in the past, it makes it anxiety inducing to consider being looked over by a doctor who has not only institutional power over me, but also societal privilege over me.”*	2	4.1%
	**d. Societal misogyny and/or sexism** *“There is typically an immediate power dynamic at play in the room due to gender - regardless of how the medical provider(s) act, etc. How exactly that power dynamic ends up settling in ultimately guides my decision ”*	3	6.1%
	**e. Discrimination or stigma due to race/nationality** *“I’m not optimistic about empathetic treatment from unknown health professionals considering my sexual orientation and my nationality”*	1	2.0%
		12	**24.5%**

No, SOGI does not influence FBSE decision (n = 112)

Yes, SOGI does influence FBSE decision (n = 49)

**Table 2:** Question 2 subthemes and exemplary quotes. *“In your opinion, what can medical providers and staff do to improve the experience of a full body skin exam for LGBTQ+ patients?”*

Suggestion	Patient Quotes
**1. The clinical environment**	
Provide gowns	*“Provide gowns for patients once they undress.”*
*Have chaperones*	*“I don’t care who you are, how you identify, have a chaperone in the room.”*
Incorporate visible displays of inclusivity	*“As small as it may sound, having a pride flag somewhere visible to patients is an easy way to let us know it is a safe space.”* *“State in their office walls, on their website, and all visible places they are lgbtq friendly.”*
Include gender identity on forms and train staff on using it	*“Doctors need to have space on their forms for gender identity information AND pay attention to it.”*
Employ more queer physicians and staff	*“Have more queer physicians.”* *I just don’t feel comfortable with straight dermatologists because they don’t seem to know what my risks or concerns are.”*
Let patients meet and choose clinicians before care	*“Ensure patient can meet and choose the doctor they trust.”*
	
**2. General tips**	
Avoid inappropriate questions about sexual orientation when irrelevant	*“Not asking questions related to their sexual Orientations.”* *“The gender identity, the sexual orientation and the gender expression are irrelevant for the medical provider and staff to do their job. All those three characteristics above mention are part of people’s private life.”*
Use correct pronouns and language, don’t use triggering language (e.g. “lay down”)	*“I believe they can be more inclusive in language of pro nouns.”* *“Even just understanding basic trans terms like pronouns and being respectful.”*
Do not make assumptions about anatomy	*“[Doctors] also need to stop assuming that i have a penis and testicles because i have a beard. i don’t care if people look at my body, but when they clearly don’t know what to expect it becomes very uncomfortable.”*
Offer SGM-specific care	*“Treat/offer guidance on trans-specific issues such as top surgery scar issues .”*
Recognize and reduce patien’s anxiety	*“Realize we may be very anxious about doing it in the first place.”* *“Acknowledge it may be uncomfortable and ask how to make it comfortable.”*
Treat SGM patients with equal care as non-SGM patients	*“Treat us like anyone else.”* *“Having had so many bad experiences with straight doctors, I am always afraid when I get treated. ALWAYS. I am always on guard.”*
	
**3. Before the exam**	
Ask relevant questions about gender and sexuality before exam, while dressed	*“Meet with patients before the exam & while dressed. Asking patient how their name & if the patient has any questions or concerns.”*
Do not put burden on patient to bring up sex/gender if it is relevant	*“I want the doctor to ask about sexual health concerns during a fullbody exam (not necessarily a routine visit). I don’t want to be the one to have to mention it.”*
Elicit patient concerns first to plan what is relevant to ask about and examine	*“How I have sex and who I have sex with [consenting adults] has NOTHING to do with my skin health. that said I didnt let on I am gay due to history of poor treatment by others.”* *“If he/she/they could just say, “now that we’ve finished this part of the exam, I’d like to ask about sexual health. Are you comfortable with having me examine your genitals for any growths or rashes?”* *“I also really appreciate when doctors say, “what have we not discussed today that is still on your mind?” Or, “What other questions or concerns do you have today?”*
Elicit trauma or trigger history from patient	*“Ask patients if there is anything they’d like the doctor to know about in advance, and list to the patient several examples out loud, including body modifications or alternative sex practices.”*
Believe the patient, feel genuine empathy	*“I imagine the doctor is hoping I will not bring it up. I feel like I’m just making them uncomfortable because they don’t know how to treat gay people or they don’t want to.”*
Ask permission to examine instead of telling the patient what will be done	*“Ask about language and ask permission instead of just saying it needs to be done.”*
	
**4. During the exam**	
Continuous consent (not “one and done”)	*“Continuously ask for consent and explain what they are doing and why.”*
Keep conversation flowing	*“Ask for comfort, keep conversation flowing.”*
Narrate the rationale for each step before and during touch	*“Continuously… explain what they are doing and why.”* *“Say exactly where on the body you are moving during the exam, not before.”* *“ For example ‘I’m now going to move toward this area or I’m going to look at your back’.”*
Conduct structured, systematic history and exam	*“I asked for genital skin to be examined; the doctor said to have it looked at by my gynecologist.”* *“When doctors are just going through a list of things to check and ask their patients, it feels comfortable and systematic.”*
Re-affirm allyship	*“Something like, “thanks for sharing that with me, I support and treat many LGBTQ patients and I hope you know you can ask me anything and I will do my best to support you and treat you.”*
	
**5. Physician Education**	
*Trauma informed care, language, & gender dysphoria*	*(“have staff take sensitivity workshops and trainings so as not to use phrases like “lay down” or other triggering phrases of sexual trauma.”)*
Improve knowledge and familiarity with SGM experiences	*“I feel they don’t know how to treat LGBTQ+ patients or don’t want to.”* *“Just be knowledgeable about how our experiences can impact getting care, especially care that is specialized for each person.”*

## Data Availability

The raw data supporting the findings of this study are available from the corresponding author upon reasonable request. De-identified qualitative data will be shared where consistent with institutional review board (IRB) approval, participant consent, and applicable privacy and ethical requirements.
